# Cardiac Complications in Cancer Patients Following Chemotherapy and Radiotherapy

**DOI:** 10.7759/cureus.88372

**Published:** 2025-07-20

**Authors:** Md. Zillur Rahman Bhuiyan, Harisul Hoque, Sarwar Alam

**Affiliations:** 1 Department of Clinical Oncology, Bangladesh Medical University, Dhaka, BGD; 2 Department of Cardiology, Bangabandhu Sheikh Mujib Medical University Hospital, Dhaka, BGD

**Keywords:** cancer radiotherapy, cardiac complications, chemotherapy-related toxicity, critical care cardiology, radiation and clinical oncology

## Abstract

Background: Cardiac complications are critical concerns in the management of cancer patients, particularly following chemotherapy and radiotherapy. These treatments, while effective against cancer, pose significant risks to cardiovascular health, potentially leading to conditions such as cardiomyopathy, arrhythmias, and coronary artery disease.

Objective: This study investigates the prevalence and nature of cardiac complications among cancer patients in Bangladesh who have undergone chemotherapy, radiotherapy, or both.

Methodology: This prospective cross-sectional observational study was conducted from April 2023 to May 2024 at the Bangladesh Medical University (BMU) in Dhaka, Bangladesh. A total of 339 cancer patients, aged over 18, who received chemotherapy, radiotherapy, or both, were included. Patients were evaluated for cardiac complications during and after treatment using clinical assessments and diagnostic tests, while the clinical indicators were measured (e.g., ECG, ECHO, biomarkers). Data were analyzed using IBM SPSS Statistics for Windows, Version 23 (Released 2015; IBM Corp., Armonk, New York, United States), with statistical significance set at a *P-*value <0.05. Clinical features and cardiac complications were observed among the patients undergoing chemotherapy and radiotherapy.

Results: The mean age of participants was 52.4 years. Cardiac complications were observed in a significant proportion of patients. Statistically significant increases in hypertension, diabetes, and levels of cardiac biomarkers (Troponin-I, NT-proBNP, CK-MB) were noted post-treatment compared to pre-treatment values (*P* < 0.001). Specifically, 83 (24.5%) patients developed hypertension, 70 (20.6%) diabetes, and notable elevations in cardiac biomarkers were recorded, indicating heightened cardiac risk.

Conclusion: The study highlights a substantial burden of cardiac complications in cancer patients receiving chemotherapy and/or radiotherapy in Bangladesh. These findings underscore the need for integrated oncologic and cardiac care to monitor and manage cardiovascular health in cancer patients. Further research is necessary to develop targeted interventions to mitigate these risks in the Bangladeshi population.

## Introduction

Cardiac complications represent a significant concern in the comprehensive care of cancer patients, posing substantial challenges to both treatment outcomes and patient well-being. These complications can arise from the cancer itself or as adverse effects of various cancer therapies, including chemotherapy, radiotherapy, and immunotherapy [1]. Among the myriad of cardiac complications encountered, cardiomyopathy, arrhythmias, pericarditis, myocardial infarction, heart failure, valvular heart disease, and coronary artery disease stand out as prominent concerns, each presenting unique clinical manifestations and management considerations [2]. While advances in cancer treatment have significantly improved survival rates, the growing recognition of cardiac complications underscores the importance of a multidisciplinary approach to patient care, integrating oncology and cardiology expertise to optimize treatment strategies and mitigate cardiovascular risks.

In Bangladesh, cancer is a significant public health issue with an increasing incidence rate. According to the International Agency for Research on Cancer (IARC), Bangladesh had an estimated 156,775 new cancer cases and 108,990 cancer-related deaths in 2020. The most common types of cancer include breast, head and neck, cervical, and lung cancers [3].

The treatment landscape in Bangladesh includes chemotherapy, radiotherapy, or a combination of both. However, access to these treatments is uneven, with most facilities located in urban areas, which poses challenges for rural populations [4,5]. According to the WHO, there are fewer than 21 radiation oncologists per 10,000 cancer patients in Bangladesh, highlighting a significant gap in the availability of specialized care [6,7].

Challenges in accessing treatment and disparities in healthcare resources contribute to the burden of cancer in the country. Efforts to address these issues include improving the distribution of healthcare services and increasing public awareness of cancer prevention and early detection. Initiatives such as the HPV vaccination campaign aim to reduce the incidence of cervical cancer, one of the most prevalent cancers among Bangladeshi women [8]. Overall, addressing the cancer burden in Bangladesh requires a multifaceted approach, including enhancing healthcare infrastructure, improving access to treatment, and promoting preventive measures through public health campaigns and policy interventions.

Cardiac complications in cancer patients following chemotherapy, radiotherapy, or both can have significant impacts on morbidity and mortality rates. Chemotherapy drugs, particularly anthracyclines like doxorubicin, can damage heart muscle cells, leading to cardiomyopathy [9]. This condition reduces the heart's ability to pump blood effectively, potentially resulting in heart failure. Chemotherapy drugs such as cisplatin and targeted therapies like trastuzumab (Herceptin) can disrupt the heart's electrical system, causing arrhythmias. These irregular heart rhythms, including atrial fibrillation, can increase the risk of stroke and other complications [10].

Inflammation of the pericardium, the membrane surrounding the heart, can occur as a result of chemotherapy drugs like cyclophosphamide or radiotherapy to the chest area [9]. Pericarditis can cause chest pain and other symptoms. Chemotherapy and radiotherapy can damage blood vessels and increase the risk of coronary artery disease, leading to myocardial infarction. Patients may experience chest pain, shortness of breath, and other symptoms [10]. On the other hand, chemotherapy-induced cardiotoxicity can weaken the heart muscle over time, leading to heart failure. Symptoms may include fatigue, swelling in the legs, and difficulty breathing [9,10].

Some chemotherapy drugs, such as doxorubicin, daunorubicin, and epirubicin, can damage heart valves, leading to valvular heart disease. This condition affects the flow of blood through the heart and may require surgical intervention [11].

Radiation therapy to the chest can increase the risk of coronary artery disease by damaging the blood vessels supplying the heart [12,13]. This can lead to chest pain (angina) and other symptoms of heart disease [14,15]. Management of these cardiac complications typically involves close monitoring, lifestyle modifications, medications to manage symptoms and prevent further damage, and in some cases, interventions such as angioplasty or surgery [16-18]. It is essential for healthcare providers to carefully assess cardiac risk factors before initiating cancer treatment and to monitor patients closely throughout their treatment course to minimize the risk of cardiac complications [12,13,15].

In such a case, the aim of this study is to find out the cardiac complications in cancer patients following chemotherapy, radiotherapy, or both chemotherapy and radiotherapy in Bangladesh.

## Materials and methods

This prospective observational study was conducted at the Department of Clinical Oncology and Cardiology of Bangladesh Medical University (BMU), Dhaka, Bangladesh from April 2023 to May 2024. A total of 339 histologically proven cancer patients treated with either chemotherapy or radiotherapy or both and post-treated cases who developed any sort of cardiac involvement were enrolled in this study after fulfilling the inclusion and exclusion criteria. Samples were collected consecutively by purposive sampling techniques. Patients of any sex aged more than 18 years with cancer treated with radiotherapy or chemotherapy or with both were included in the study. Diagnosed cases of cancer patients having cardiac co-morbidities, seriously ill patients, pregnant women, and patients who did not give consent were not included in the study.

Follow-ups were done during and after radiotherapy or chemotherapy or both. Data was collected, compiled, and tabulated according to key variables and functional assessment scoring. The analysis of different variables was done according to standard statistical analysis. Quantitative data have been expressed as frequency and percentage, and quantitative data have been expressed as mean and standard deviation. Quantitative data was analyzed by Student's t-test and qualitative data by the chi-square test. IBM SPSS Statistics for Windows, Version 23 (Released 2015; IBM Corp., Armonk, New York, United States) was used to process and analyze the data. For all analyses, the significance level was set at 0.05, and a P-value <0.05 was considered statistically significant.

## Results

The mean (±SD) age of the participants of the study was 52.4 (10.07), where the minimum age of the participants was 25 and the maximum age of the participants was 75. There was almost an equal number of male and female participants. The residential status of the participants was also similarly distributed, where 174 (51.3%) participants belonged to the urban areas, and the rest belonged to rural areas. Among the occupations of the participants, most of the female participants were housewives, so this was the highest occupational status of the participants, which is 163 (48.1%), followed by business, which is 68 (20.1%), and farming among 57 (16.8%) participants. According to the socio-economic status of the participants, 227 (67%) of the participants belonged to the middle class. The number of family members varied between four and 12 members in the family of the participants. About 206 (60.8%) participants had six family members, followed by 122 (36%) participants having four family members (Table 1).

**Table 1:** Socio-demographic characteristics of the participants

Characteristics	Frequency	Percentage
Age		
25-34	13	3.8
35-44	59	17.4
45-54	134	39.5
55-64	77	22.7
65 and above	56	16.5
Sex		
Male	169	49.9
Female	170	50.1
Residential status		
Rural	165	48.7
Urban	174	51.3
Occupational status		
Service	27	8
Business	68	20.1
Labor	24	7.1
Farmer	57	16.8
Housewife	163	48.1
Socio-economic status		
Lower	60	17.7
Middle	227	67
Upper	52	15.3
Number of family members		
Four or less	122	36
Five to Nine	206	60.8
Ten and more	11	3.2

Among the participants, 102 (30.1%) were smokers, 136 (40.1%) consumed betel nut, and three (0.9%) participants consumed alcohol. Regarding family history of cancer, 96 (28.3%) participants reported a positive family history, and 68 (20.1%) were unsure. Among the pre-treatment co-morbidities, 83 (24.5%) participants had hypertension (HTN), 70 (20.6%) had diabetes mellitus (DM), and 40 (11.8%) had renal disease (Table 2).

**Table 2:** Clinical characteristics of the participants HTN: Hypertension; DM: diabetes mellitus

Characteristics	Frequency	Percentage
Smoking		
Yes	102	30.1
No	237	69.9
Betel nut		
Yes	136	40.1
No	203	59.9
Alcohol		
Yes	3	0.9
No	336	99.1
Family history of cancer		
Yes	96	28.3
No	175	51.6
Unknown	68	20.1
Having pre-treatment co-morbidities		
HTN		
Yes	83	24.5
No	256	75.5
DM		
Yes	70	20.6
No	269	79.4
Renal disease		
Yes	40	11.8
No	299	88.2

The different types of cancer that were diagnosed were Ca. Lung among 77 (22.7%), Ca. Breast among 57 (16.8%), Ca. Cervix among 33 (9.7%), Ca. Prostate among 25 (7.4%), Ca. Ovary among 24 (7.1%), Ca. Rectum among 22 (6.5%), Ca. Stomach among 20 (5.9%), Ca. Ascending colon among 15 (4.4%), Ca. Descending colon among 15 (4.4%), Ca. Pancreas among 15 (4.4%), CNS Tumor among 14 (4.1%), sarcoma among 14 (4.1%), and Ca. Caecum among eight (2.4%) participants. Among the grade of the participants suffering from cancer, nine (2.7%) participants were in grade I, 86 (25.4%) in grade II, 114 (33.6%) in grade III and 130 (38.3%) in grade IV. Treatment modality included 66 (19.5%) participants who received radiotherapy alone, 71 (20.9%) participants who received chemotherapy alone, and 203 (59.6%) participants who received both radiotherapy and chemotherapy (Table 3).

**Table 3:** Diagnosis and stage of the disease of the participants

Characteristics	Frequency	Percentage
Diagnosis		
Ca. Ascending colon	15	4.4
Ca. Breast	57	16.8
Ca. Caecum	8	2.4
Ca. Cervix	33	9.7
Ca. Descending colon	15	4.4
Ca. Lung	77	22.7
Ca. Ovary	24	7.1
Ca. Pancreas	15	4.4
Ca. Prostate	25	7.4
Ca. Rectum	22	6.5
Ca. Stomach	20	5.9
CNS Tumor	14	4.1
Sarcoma	14	4.1
Grade		
I	9	2.7
II	86	25.4
III	114	33.6
IV	130	38.3
Treatment therapy		
Radiotherapy	66	19.5
Chemotherapy	71	20.9
Both	203	59.6

There was a statistically significant difference in hypertension, DM, ECG, ECHO, Troponin-I, NT Pro BNP, and CK-MB findings of the participants in pre-treatment and data taken after six months when the participants received chemotherapy or radiotherapy, or both (P <.001). The mean (± SD) of DM, ECHO, Troponin-I, NT Pro BNP, and CK-MB increased from 5.1 (±.69) to 5.2 (±.70), 57.43 (± 9.26) to 58.95 (± 9.93), 0.76 (± 0.29) to 4.39 (± 3.09), 501.18 (± 49.75) to 528.92 (±49.48), and 20.1 (± 0.5) to 20.4 (± 0.7), respectively which were all statistically significant (P <.001) (Table 4).

**Table 4:** Pre-treatment and follow-up result of relevant cardiac investigations * P < 0.05 considered statistically significant; ** P < 0.001 considered highly significant HTN: Hypertension; DM: diabetes mellitus

Characteristics	Pre-Treatment	At Six Months	P-Value	Statistical Test Used
HTN	1.2 (0.2)	1.3 (0.3)	< .02*	McNemar’s Test
DM	5.1 (±.69)	5.2 (±.70)	< .001**	McNemar’s Test
ECG	1.4 (± 0.5)	1.5 (± 0.6)	< .01*	Paired t-test
ECHO	57.43 (± 9.26)	58.95 (± 9.93)	< .001**	Paired t-test
Troponin-I	0.76 (± 0.29)	4.39 (± 3.09)	< .001**	Paired t-test
NT Pro-BNP	501.18 (± 49.75)	528.92 (±49.48)	< .03*	Paired t-test
CK-MB	20.1 (± 0.5)	20.4 (± 0.7)	< .001**	Paired t-test

## Discussion

Specific studies focusing on cardiac complications in cancer patients following chemotherapy, radiotherapy, or both in South Asia may be limited. However, some studies from the broader Asian region, which may include South Asian populations, have highlighted certain cardiac complications.

A study found that anthracycline-based chemotherapy was associated with a significant risk of cardiotoxicity, including cardiomyopathy, in breast cancer patients [18]. Another study from Pakistan reported that cardiomyopathy was observed in a subset of pediatric cancer patients treated with anthracyclines [19]. A retrospective study in Taiwan found that cancer patients treated with cisplatin-based chemotherapy had an increased risk of developing arrhythmias, particularly atrial fibrillation [20]. Data specific to South Asian populations regarding pericarditis and myocardial infarction following cancer treatment are limited. However, it is known that certain chemotherapy drugs and radiotherapy can increase the risk of these complications. A study observed a significant incidence of heart failure in cancer patients receiving anthracycline-based chemotherapy, particularly in those with pre-existing cardiovascular risk factors [21]. These studies provide insights into the potential cardiac complications faced by cancer patients undergoing chemotherapy and radiotherapy in South Asia. However, further research specifically focusing on South Asian populations is needed to better understand the prevalence, risk factors, and outcomes associated with cardiac complications in this region.

The present study found a high prevalence of cardiac complications in cancer patients undergoing chemotherapy, radiotherapy, or both in Bangladesh. These findings are consistent with extensive research from around the world demonstrating the cardiotoxic effects of cancer therapies. A review of 12 articles among women with breast cancer found that those receiving left-sided radiotherapy had a 29% increased risk of developing coronary heart disease and a 22% increased risk of cardiac death compared to those receiving right-sided radiotherapy [22]. The risk started increasing within the first decade after radiotherapy for coronary heart disease and from the second decade for cardiac mortality. Radiotherapy to the chest area for cancers like lung, esophagus, breast cancer, lymphoma, and stomach can lead to increased non-cancer mortality, primarily due to cardiac causes [23]. Major risk factors include older radiation techniques, high radiation doses over 30-35 Gy, concurrent cardiotoxic chemotherapy like anthracyclines, larger irradiated heart volumes, anterior or left-sided chest radiation, younger age at treatment, and presence of cardiovascular risk factors [24].

A study by Darby et al. reported a linear 7.4% increase in relative risk of major coronary events per Gy increase in mean heart radiation dose, independent of cardiac risk factors, with no safe threshold dose [25]. The increased risk started shortly after radiation exposure and continued for at least 20 years. Radiation can damage all cardiac structures, including accelerating atherosclerosis and causing coronary artery disease decades later, microvascular injury, reducing myocardial capillary density, and valve endothelial injury, causing valve disease [26]. These mechanisms are worsened by concurrent chemotherapy and cardiovascular risk factors. The estimated incidence of radiation-induced heart disease is 10-30% after 5-10 years post-treatment, with a 1.7 to 2-fold increase in cardiovascular mortality in radiated patients [27]. The incidence is higher in lymphoma patients compared to breast cancer patients, approaching 50%. For lung cancer, radiotherapy increases cardiac mortality risk by nearly 30% [28]. Management follows the same treatment options as non-radiation-induced heart disease, both medical and surgical. Transcatheter aortic valve replacement has shown lower mortality than surgical aortic valve replacement for radiation-induced valvular disease. Coronary revascularization outcomes are similar to non-radiation cases.

This article has some limitations. The absence of a non-cancer control group or patients not receiving chemotherapy or radiotherapy limits the ability to attribute cardiac complications solely to cancer treatment, as other confounding factors may also play a role. Some variables, such as family history of cancer and lifestyle risk factors, were self-reported, which could introduce recall bias and affect data accuracy. The inclusion criteria required patients to be actively receiving or have completed treatment, possibly excluding patients with severe complications or those lost to follow-up, thus underrepresenting severe cases.

## Conclusions

In conclusion, this study highlights the significant prevalence of cardiac complications in cancer patients following chemotherapy and radiotherapy, particularly in the form of heart failure, arrhythmias, and coronary artery disease. To improve patient outcomes, we recommend integrating cardio-oncology care into routine cancer treatment protocols. Risk-adapted monitoring should be implemented, with more frequent cardiovascular assessments for patients with higher susceptibility, such as those undergoing anthracycline-based therapies or with pre-existing cardiovascular conditions.

Furthermore, our study suggests that local policy adjustments are necessary to incorporate routine cardiac screening and prevention into clinical practice for cancer patients. This could involve creating or updating guidelines for cardiotoxicity surveillance, alongside ensuring proper training for oncologists and cardiologists in collaborative care models. Future research is needed to further investigate the long-term effects of cancer therapies on cardiac health and to refine risk stratification models. With continued focus on these areas, it will be possible to mitigate the cardiac risks faced by cancer survivors and enhance their overall quality of life. Incorporating these recommendations into local healthcare systems will help ensure a more comprehensive approach to cancer care, addressing both the immediate and long-term health of patients.

## Appendices

The operational definitions outlined in this study provide a structured framework for measuring and categorizing the core variables under investigation, ensuring clarity and consistency throughout data collection and analysis. The study defines a cancer patient as any individual diagnosed histologically with cancer and confirmed by a pathologist, who is either undergoing or has previously undergone chemotherapy, radiotherapy, or both. This comprehensive definition ensures that only those with medically confirmed malignancies and relevant exposure to cancer treatments are included in the analysis, enhancing the internal validity of the study [13].

Chemotherapy is operationalized as the administration of cytotoxic drugs specifically aimed at destroying malignant cells. It is quantified based on dosage (mg/m²) and duration (number of treatment cycles), enabling assessment of both intensity and length of exposure to these agents [14]. Similarly, radiotherapy is defined as treatment using ionizing radiation to target cancerous tissues, with doses recorded in Gray (Gy), taking into account total delivered dose and fractionation schedules. This precise measurement allows comparison across different radiotherapy regimens and facilitates correlation with subsequent cardiac outcomes.

To examine the potential cardiovascular consequences of cancer therapy, the study includes a detailed categorization of cardiac complications. These complications are identified using objective clinical tests and imaging studies. Cardiomyopathy, defined as a disease of the heart muscle that impairs its ability to pump blood effectively, is a central concern, especially given the cardiotoxic potential of many chemotherapeutic agents. Arrhythmias, or irregular heart rhythms, are detected through electrocardiography (ECG), which provides a non-invasive method for identifying electrical abnormalities in heart function. Pericarditis, defined as inflammation of the pericardial sac surrounding the heart, is diagnosed based on clinical symptoms like chest pain and supported by imaging techniques such as echocardiography. Myocardial infarction (MI) is defined as irreversible necrosis of heart muscle due to ischemia and is confirmed through the elevation of cardiac biomarkers like Troponin-I and characteristic ECG changes [13,15]. Heart failure is also considered, described as a clinical syndrome arising from impaired cardiac filling or ejection. It is diagnosed based on symptoms such as dyspnea and supported by elevated NT-proBNP levels, which are sensitive indicators of heart failure status.

The research also considers a range of demographic variables. Age is measured in completed years, while sex is categorized into male, female, or other, acknowledging gender diversity. Residence is classified as rural or urban, allowing exploration of geographic disparities in healthcare access or outcomes. Comorbidities such as hypertension (defined as BP >140/90 mmHg), diabetes mellitus (Fasting Blood Sugar ≥126 mg/dL), and renal disease (e.g., serum creatinine >1.5 mg/dL) are included due to their potential confounding effects on cardiac outcomes and treatment tolerance [14]. The presence of a family history of cancer, whether self-reported or documented, is also recorded to explore any hereditary predisposition to cancer development.

The inclusion of biomarkers adds a quantitative dimension to the assessment of myocardial injury and cardiac dysfunction. Troponin-I levels above 0.04 ng/mL are indicative of myocardial injury, while CK-MB levels exceeding 6 ng/mL suggest cardiac muscle damage. NT-proBNP, a biomarker strongly associated with heart failure, is interpreted using age-specific cutoffs. These biochemical parameters serve as sensitive and specific indicators of cardiac health and are crucial for early detection of treatment-related cardiotoxicity.

Hypertension (HTN) is further operationalized using a five-stage categorization, ranging from normal blood pressure to hypertensive crisis, based on systolic and diastolic blood pressure readings. This detailed classification allows for nuanced assessment of cardiovascular risk among cancer patients. Similarly, ECG results are categorized from 1 to 5 based on detected abnormalities, enabling standardized comparison across patients [15].

Another critical diagnostic tool included is echocardiography (ECHO), specifically focusing on left ventricular ejection fraction (LVEF). The classification ranges from normal function (55-70%) to severely reduced function (<30%), offering a gradient for evaluating the severity of cardiac impairment. This classification enables researchers to stratify patients based on functional cardiac status, which may correlate with the intensity or duration of cancer treatment. Conditions such as mild or moderate cardiomyopathy or advanced heart failure can be directly inferred from these echocardiographic findings [16,17].

Socioeconomic status is determined by monthly family income and stratified into lower, middle, or upper classes based on nationally accepted income brackets. This allows for exploration of economic disparities in access to cancer care, adherence to treatment, or cardiovascular outcomes. Cancer staging, using the TNM classification or local protocols, offers a clinical estimate of disease progression and is essential for contextualizing treatment intensity and potential side effects.

Finally, follow-up duration, defined as the time from treatment initiation to the latest assessment, is recorded in months. This temporal variable is crucial in determining the latency of cardiac complications and assessing long-term outcomes. Extended follow-up periods are particularly important in capturing late-onset cardiotoxicity, which may not be apparent during or immediately after cancer therapy.

Collectively, these operational definitions provide a comprehensive and standardized basis for the study, ensuring precision and consistency in the measurement of variables. They also facilitate replication and comparison with other studies and are aligned with established clinical guidelines and research literature. The integration of diagnostic thresholds, clinical categorizations, and objective biomarkers strengthens the study's methodological rigor and supports the accurate assessment of the relationship between cancer therapies and cardiovascular outcomes.
